# Pure Endovascular Management of an Arteriovenous Malformation and an Aneurysm Both Supplied by Anterio-Inferior Cerebellar Artery: A Case Report and a Review of Literature

**DOI:** 10.3389/fneur.2017.00382

**Published:** 2017-08-02

**Authors:** Hassan A. Khayat, Fawaz Alshareef, Abdulrahman Alshamy, Abdulrahman Algain, Essam Alhejaili, Omar Alnabihi, Saeed Alzahrani, Ruediger Stendel

**Affiliations:** ^1^King Abdullah International Medical Research Center (KAIMRC)/Department of Neurosurgery-King Abdulaziz Medical City (KAMC), Ministry of National Guard Health Affairs (MNG-HA), Jeddah, Saudi Arabia; ^2^King Abdullah International Medical Research Center/Department of interventional radiology-King Abdulaziz medical city (KAMC), Ministry of National Guard Health Affairs (MNG-HA), Jeddah, Saudi Arabia; ^3^King Abdullah International Medical Research Center/College of Medicine – King Saud bin Abdulaziz University for Health Sciences (KSAUHS), Ministry of National Guard Health Affairs (MNG-HA), Jeddah, Saudi Arabia

**Keywords:** arteriovenous malformation, aneurysm, anerior inferior cerebellar artery, endovascular approach, posterior fossa

## Abstract

**Background and importance:**

The tendency of posterior fossa arteriovenous malformations (pfAVM) to develop associated aneurysms (AA) is a well-known phenomenon with an increased total risk of rupture. Most pfAVM and AA develop in the territory of the posterior inferior cerebellar artery while the involvement of the anterior inferior cerebellar artery (AICA) is extremely rare. We describe an unusual case of an arteriovenous malformation (AVM) supplied by the AICA with a “proximal” AA. This unique combination of vascular lesions has been reported in only four cases so far, limiting the available experience that can safely guide the therapeutic intervention.

**Clinical presentation:**

This study describes a 59-year-old female presented with a subarachnoid hemorrhage, Hunt and Hess grade 4. Angiography demonstrated an AVM fed mainly by the right AICA and draining superficially into the transverse sinus (Spetzler–Martin grade II). In addition, there was a ruptured proximal AICA aneurysm. An endovascular approach was chosen to coil the aneurysm and obliterate the AVM using ONYX in a multi-staged process. The patient recovered well without residual deficit at 6-month follow-up.

**Conclusion:**

To the best of our knowledge, this is the first report describing a proximal AICA aneurysm and AVM treated by endovascular means. The outcome was very good, considering the technically demanding location. All previously reported cases with exactly similar lesions were managed surgically, with inconclusive outcomes. The data presented in this study are meant to help in decision-making process for similar cases till more data are available.

## Background and Importance

Only 8–12% of intracranial aneurysm and 5–15% of arteriovenous malformations (AVMs) occur in the posterior circulation (1, 2). The association between an AVM and aneurysm of the same artery is a well-known phenomenon, reported in 2.8–9.3% of all AVMs (3). Notably, posterior fossa AVM’s have an increased tendency to develop associated aneurysms compared to AVMs of the anterior circulation (2), and mostly involve the posterior inferior cerebellar artery. The involvement of the anterior inferior cerebellar artery (AICA) is extremely rare (2). Reviewing the literature, only 16 cases of coincident AICA aneurysm and AVM have previously been reported (1, 3–9). Proximal aneurysms, exactly as the index case of this study, occurred in only four cases (7–9).

Herein, we describe an extremely rare case of a cerebellar AVM supplied by the AICA and a proximal aneurysm fed by the same artery, which was managed completely using an endovascular approach. In contrast, all previously reported cases with similar lesion complex were managed surgically. This study provides justifications why endovascular approach was chosen. We also provide a comprehensive review of similar cases reported in the literature.

## Methods

This study has been approved by the independent ethics committee (Institutional Review Board) at the King Abdullah International Medical Research Center—Jeddah, Saudi Arabia. A written informed consent was obtained from the index case of this study to participate and publish the paper once the study is done. All related information utilized in this paper was obtained through chart review, patient history, and clinical examination.

## Clinical Presentation

**Figure 1:**
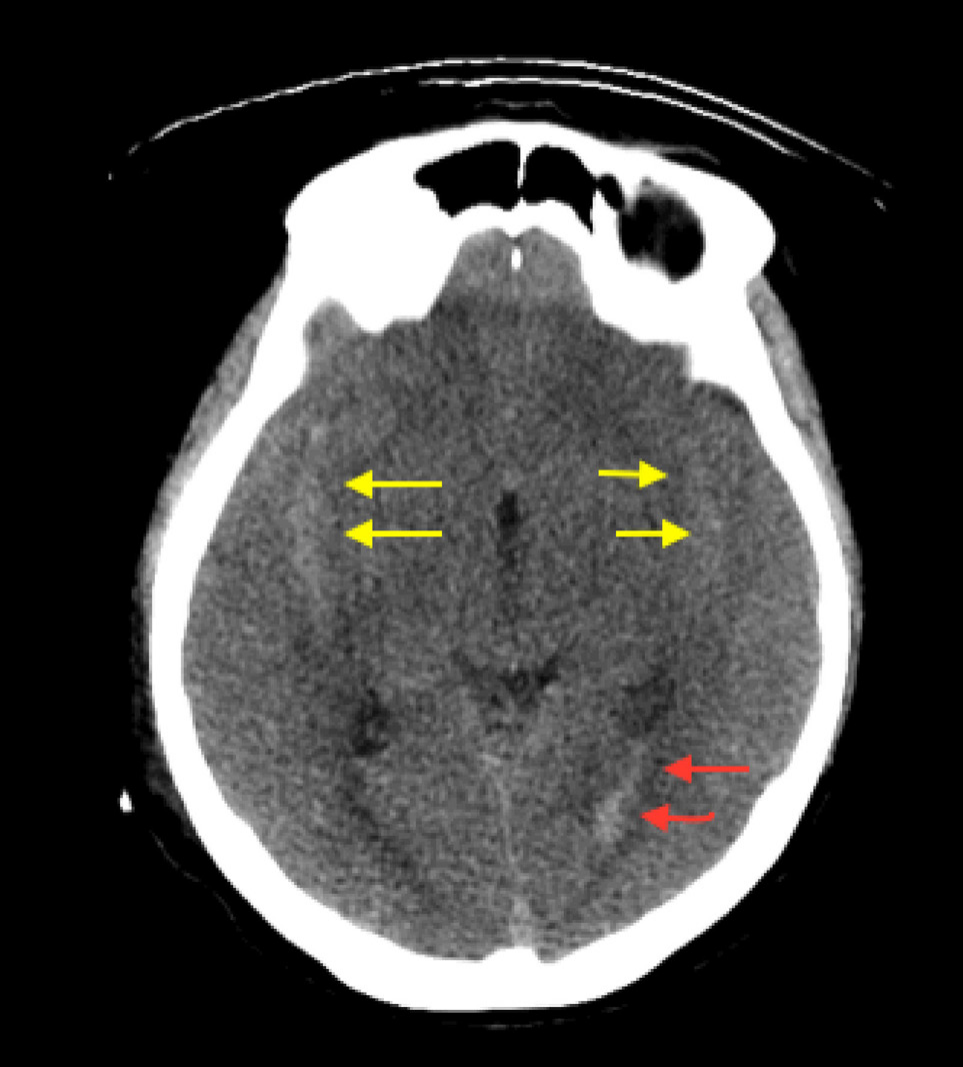
Axial section CT demonstrating diffuse brain edema evident by effacement of cortical sulci and the subarachnoid hemorrhage in both Sylvian fissures (yellow arrows) with seeding into the ventricular system (red arrows).

**Figure 2:**
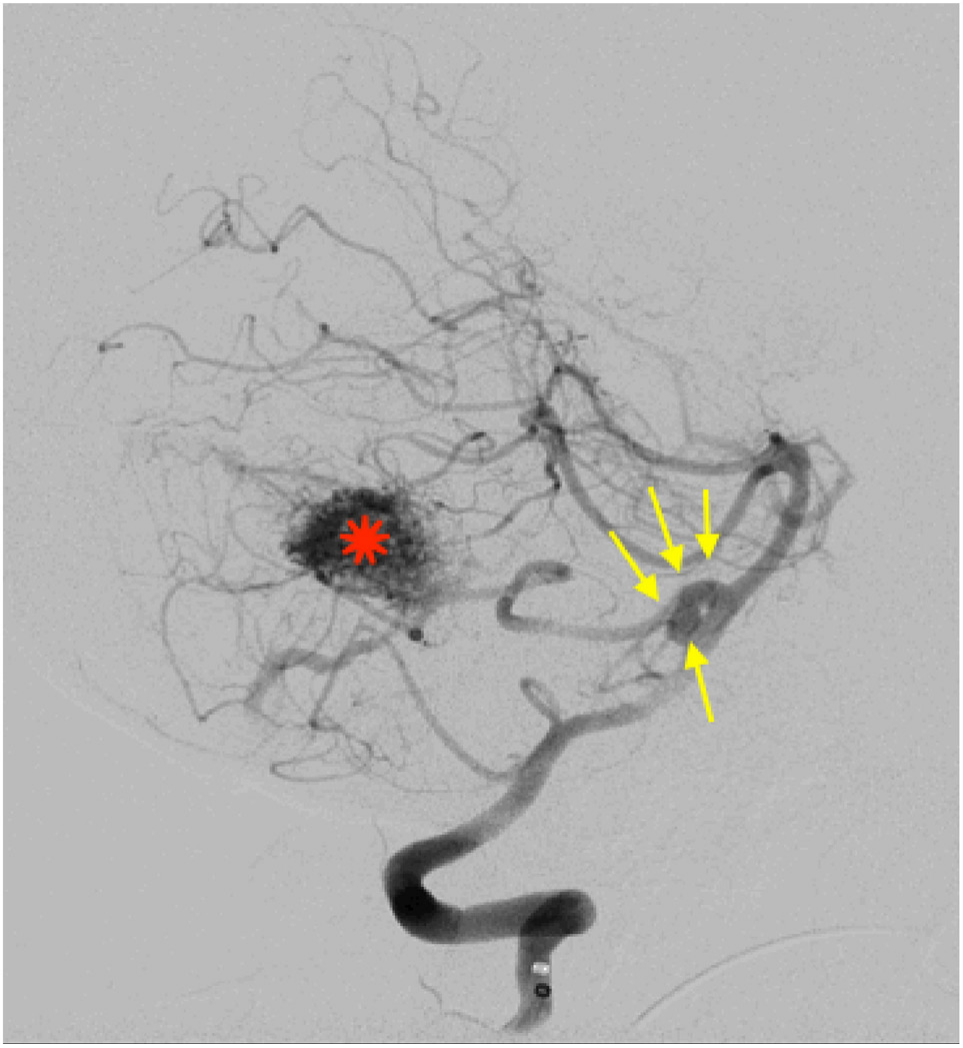
Lateral view of a right vertebral angiography revealing a ruptured proximal aneurysm (7 mm × 3 mm) at the origin of AICA (yellow arrows). Distally on the same trunk, a nidus of an arteriovenous malformation is demonestrated (diameter = 2.6 cm) (red star).

A 59-year-old Saudi woman presented with a deteriorated level of consciousness after 2 days of severe headaches and multiple episodes of vomiting. A non-enhanced brain CT scan showed a subarachnoid hemorrhage (SAH) (Hunt and Hess grade 4–5), intraventricular hemorrhage, and generalized brain edema (Figure 1). Angiographic series delineated a cerebellar AVM with a nidus of 14 mm × 20 mm in diameter, situated on the post meatal segment of AICA and draining superficially into the transverse sinus (Spetzler–Martin grade II). The nidus was primarily supplied by the AICA, which developed a pre-nidal saccular aneurysm measuring 7 mm × 3 mm (Figure 2). The aneurysm was the source of bleeding.

**Figure 3:**
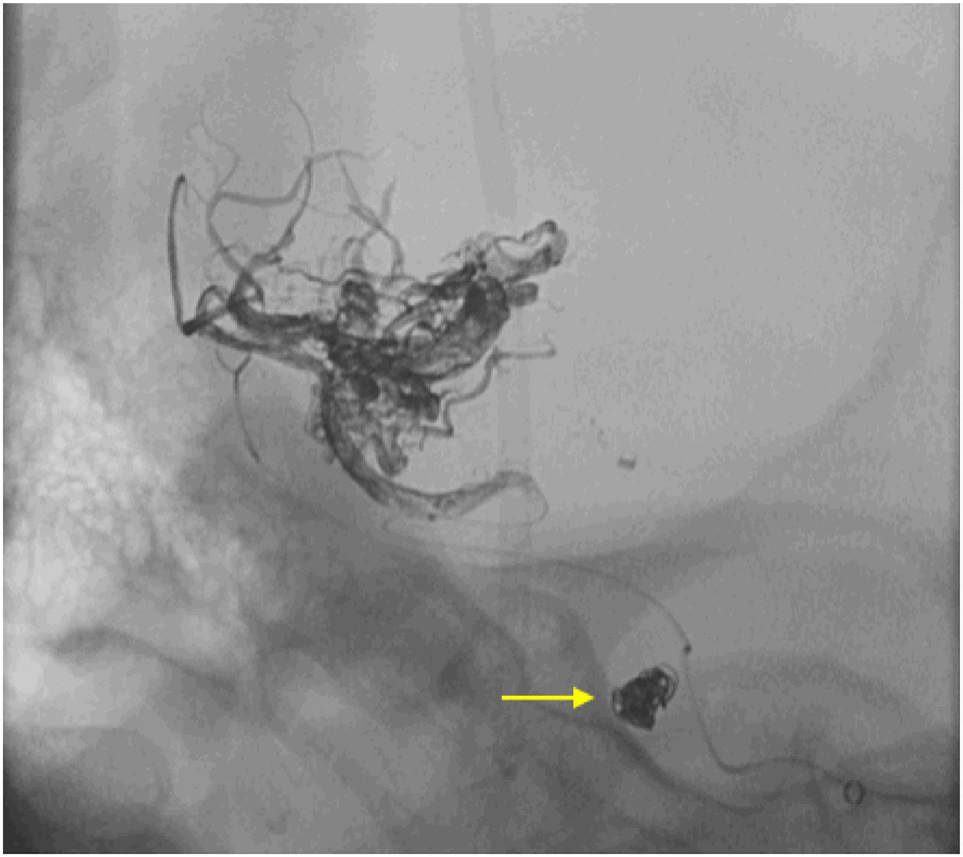
Final run X-Ray demonstrating the position and configuration of the platinum coils inside the sac of the aneurysm (yellow arrow) as well as the ONYX filling the vascular channels of the arteriovenous malformation after the procedure.

Considering the size and the proximal location of the aneurysm, along with the low grade of the AVM, as well as the generalized brain edema and SAH, an endovascular approach was decided. The aneurysm was accessed using a 5-French wire-guided catheter inserted, within a 6-French sheath, into the right femoral artery and progressed under fluoroscopy road-map guidance. Coiling of the aneurysm was achieved using three platinum-made, detachable coils (3 mm × 8mm, 2 mm × 8mm, 1.5 mm × 4mm). During the same procedure, the distally located AVM was embolized using a liquid-based suspension consisting of Ethylene–Vinyl–Alcohol copolymers (ONYX), applied through a micro-catheter (APOLLO 38), with a 5-cm detachable tip. Seventy percent of the AVM was obliterated initially and then completed after 6 months. The final series of X-Ray (Figure 3) confirmed the proper positioning of the coils within the aneurysmal sac and occlusion of the AVM. The patient recovered well upon rehabilitation with no evidence of rebleeding or focal neurological deficits at 6-month follow-up. She is fully mobile and independent with a Glasgow outcome score of 4–5.

## Discussion

### AVM with AA: The Theory of Hemodynamic Interaction

The therapeutic intervention for AVMs with associated pre-nidal aneurysms is complex and multifaceted. A key concept to consider is that both lesions are equally exposed to the same high-pressure arterial flow simultaneously. Therefore, the hemodynamic stress is distributed on both lesions in a way determined by function of the vascular capacitance of each lesion (2). Thus, treatment of either lesion alone can cause the other one to rupture, owing to the hemodynamic alteration that occurs after the procedure (3). Some authors have, therefore, recommended treating the symptomatic lesion first, and subsequently securing the other lesion during the same procedure (3). In this study, we attempted to secure the ruptured aneurysm first by coiling, followed by the embolization of the AVM with no adverse outcomes observed.

### Approach Consideration: The Decision to Coil or to Clip

The decision to coil or to clip an aneurysm has been debated for many years. Unfortunately, no simple and straightforward answer can be provided. However, clipping of AICA aneurysm presents a magnificent challenge because of its small caliber, torturous course and proximity to eloquent areas and multiple cranial nerves (3). Complications are reported to occur after this procedure in up to 60% of cases, even when performed by experienced surgeons (3). Classically, clipping of AICA aneurysms is usually done through a retrosigmoid approach as it provides an adequate surgical corridor exposing the entire area of the AICA (8). However, in a state of acute cerebellar edema, as seen with the index case, more laborious retractions of the cerebellum would be necessary to achieve an adequate exposure. This can potentially jeopardize the nearby vital structures such as the cranial nerves V–VIII, the brainstem, and the cerebellum itself (8).

On the other side, endovascular coiling has been in clinical use since 1995 (8). In a single-center study, five out of 34 patients diagnosed with isolated AICA aneurysms were treated with an endovascular approach, which was successful in 3/5 (8).

As the evidence is somehow still conflicting, and the absolute benefit of either coiling or clipping these lesions was not clearly provided in the literature, “do no harm” was the principle of our priority. Endovascular intervention was chosen as it provides an easy access for both lesions without disturbing the integrity of nearby structures complicated by edema and SAH-related vasospasm.

### A Comparison with Similar Cases

None of the previous 16 cases of aneurysms and AVMs of AICA were treated with endovascular intervention (1, 3–9); see (Table 1). We suggest that endovascular approach, whenever affordable and technically feasible, should be considered in the treatment of an AVM and proximal aneurysm that are both fed by AICA. Supporting this, previously published data indicated that coil embolization was particularly useful for proximal AICA aneurysms associated with distal AVM and probably being the treatment of choice (1). A stronger evidence has come from a landmark multi-center study, the *ISAT* trial (10). However, it should be noted that this trial did not specifically consider the presence of associated AVMs and most of the enrolled cases were having aneurysms in the anterior circulation, not in the posterior fossa. This would create a potential bias.

**Table 1:** Summary of previously reported cases with AICA aneurysm and AVM.

Patient age/sex (reference)	Presentation	Location aneurysm/AVM	Size aneurysm/AVM	Treatment of aneurysm	Approach	Treatment of AVM	Complication
47/male (4)	SAH	Right distal/distal	7/12 mm	Clipping	Retrosigmoid	Resected	None
54/male (8)	SAH/IPH	Distal		Clipping	Retrosigmoid	Resected	Motor deficit
46/female (8)	SAH/IVH	Proximal		Clipping	Retrosigmoid	Resected	CN VII/Motor deficit
24/female (8)	SAH	Proximal		Clipping	Temporal	Resected	None
72/female (8)	SAH	Aneurysm: distal		Clipping	Transcochlear	Resected	None
17/female (3)	Headache/LLOC	Distal/distal	2/2.5cm	Clipping	Retromastoid	Not resected	None
59/female (5)	SAH	Distal/distal	Aneurysm: 3 mm × 3 mm	Aneurysm resected; AVM seemed inoperable		Not resected	None
28/female (5)	SAH	Distal/distal	Aneurysm: 4 mm × 4 mm	Resection		Resected	None
35/male (1)	SAH, IPH	Distal/distal	12/15 mm	Ligation	Retrosigmoid	Resected	Facial palsy
52/male (9)	SAH	Proximal/distal		Clipping	Rertomastoid	Not resected	Dizziness
45/male (7)		Proximal		Obliterated	Retromastoid	Extirpated	Trigeminal neuralgia
35/female (10)	SAH, CNP VIII	Distal		Clipping		Resected	None
55/male (10)	IVH, SAH	Distal/distal	Aneurysm: 2.5 mm	Trapping	Retromastoid	Not resected	None
41/male (10)	SAH, AVH	Aneurysm: distal		Clipping		Resected	Dysarthria
(6)				Clipping		Removed from CPA	Right hemiplegia
(6)				Only exploration		Not resected	AVM ruptured fetally into pons
59/female^a^	SAH/IVH	Proximal	7 mm × 3 mm/14 mm × 20 mm	Coiling	Endovascular	Embolization	None

*AICA, anterior inferior cerebellar artery; AVM, arteriovenous malformation; CPA, cerebellopontine angle; SAH, subarachnoid hemorrhage; IVH, intraventricular hemorrhage; IPH, intraparenchymal hemorrage; LLOC, low level of consciousness*.

*^a^The present study*.

The case reported here, being managed by endovascular approach, sustained an uneventful course in the immediate post interventional period as well as on 6-month follow up. No residual neurological deficit occurred and the patient was fully mobile and independent.

## Conclusion

To the best of our knowledge, this study is the first report describing a proximal AICA aneurysm and AVM treated by endovascular means. The outcome was highly promising, considering the technically demanding location and the complexity of the target lesions. In comparison, all previously reported cases with exactly similar lesions were managed surgically, with inconclusive outcomes. A collaboration of a multi-disciplinary team, including a neurosurgeon, an interventional radiologist, intensivists, and physiotherapists, is highly suggested, with potential impact on the outcome. The results of the reported case are meant to help in the decision-making process for similar cases till more data are available on this rare occasions.

## Ethics Statement

This study was carried out in accordance with the recommendations of “King Abdullah International Medical Research Center” with written informed consent from all subjects. All subjects gave written informed consent in accordance with the Declaration of Helsinki. The protocol was approved by the “independent ethics committee (Institutional Review Board—IRB) at the King Abdullah International Medical Research Center (KAIMRC)—Jeddah, Saudi Arabia.”

## Author Contributions

All authors of this manuscript have actively participated in data acquisition of this paper and they all revise and approve the final form of this paper. They are also responsible for the accuracy and integrity of this work. HK and RS participated in the design of the study. FA and AA did work on the interpretation of the data.

## Conflict of Interest Statement

The authors declare that the research was conducted in the absence of any commercial or financial relationships that could be construed as a potential conflict of interest.
